# Supplementary material for: Motivational signals disrupt metacognitive signals in the human ventromedial prefrontal cortex
Source: Commun Biol. 2022 Mar 18;5:244. doi: 10.1038/s42003-022-03197-z (PMC8933484; doi:10.1038/s42003-022-03197-z)
Supplement: Supplementary file 2 — Supplemental Information [file 42003_2022_3197_MOESM2_ESM.pdf]

Supplementary Materials

Motivational signals disrupt metacognitive signals in the human ventromedial prefrontal cortex

Monja Hoven*[1], Gina Brunner[1,2], Nina S. de Boer[1,3], Anneke Goudriaan[1,4], Damiaan Denys[1,5], Ruth van Holst‡[1], Judy Luigjes ‡[1] & Mael Lebreton‡[6,7]

[1]Department of Psychiatry, Amsterdam UMC, University of Amsterdam, Amsterdam, The Netherlands
[2]Institute of Neuroscience and Psychology, University of Glasgow, Glasgow, United Kingdom.
[3]Department of Philosophy, Radboud University, Nijmegen, The Netherlands
[4]Arkin and Jellinek, Mental Health Care, Amsterdam, The Netherlands
[5]Netherlands Institute for Neuroscience, an Institute of the Royal Netherlands Academy of Arts and Sciences, Amsterdam, The Netherlands
[6]Swiss Center for Affective Science, University of Geneva, Geneva, Switzerland
[7]Laboratory for Behavioral Neurology and Imaging of Cognition, Department of Fundamental Neurosciences, University of Geneva, Geneva, Switzerland

* Corresponding author
‡ These authors contributed equally to the manuscript

Supplementary Note 1: Full behavioral models

To assess whether our main behavioral results on confidence still hold in a full model, considering various other factors, we performed a model selection procedure of various linear mixed effect models. We used linear mixed-effects models (LMEM)– as implemented in the lmer function from the lme4 package in R (Version 1.1-12; Bates, Maechler)[1].

We iteratively built several LMEMs (Supplementary Table 1), and the final one was selected by model comparison, assessing model fit by using chi-square tests on the log-likelihood values, as well as comparison of the AIC and BIC model values (Supplementary Table 1). Model predictors were added whenever model fit was significantly improved.

The final model included fixed effects of incentive value (gain (1), neutral (0) or loss (-1)), evidence, accuracy (correct (1) or incorrect (0)), the interaction of accuracy end evidence, reaction time, and difficulty level (easy (1), medium (2), difficult (3)), as well as a random intercept and slope for the effect of incentive on confidence (model 9, see Supplementary Table 1). Satterthwaite approximations[2] were used to calculate degrees of freedom and p-value estimates for the fixed effects' regression coefficients by using the 'lmerTest' package[3] (Version 2.0-36). Visual inspection of residual plots did not reveal any obvious deviations from homoscedasticity or normality.

Final model results revealed that the significant effect of net incentive value on confidence still holds, while considering all other factors ($\beta = 0.88 \pm 0.30$, $t_{32} = 2.94$, $P = 0.006$) (Supplementary Table 2). Moreover, we found a significant effect of RT on confidence, showing that quicker choices lead to higher confidence levels ($\beta = -5.24 \pm 0.21$, $t_{4305} = -25.34$, $P < 2e-16$) (Supplementary Table 2). We also replicated that the link between confidence ratings and evidence is positive for correct and negative for incorrect responses.

**Model**	**Model notation**	**AIC**	**BIC**	**Model comp.**	χ^2	**P-value**	**Winning model**
**1**	Confidence ~ Incentive + (1|Subject)	35083	34109				
**2**	Confidence ~ Incentive + (1+Incentive|Subject)	34077	34115	1 vs. 2	10.64	0.005	2
**3**	Confidence ~ Incentive + Accuracy + (1+Incentive|Subject)	33920	33964	2 vs. 3	158.78	<0.001	3
**4**	Confidence ~ Incentive + Accuracy + Evidence + (1+Incentive|Subject)	33817	33868	3 vs. 4	104.68	<0.001	4
**5**	Confidence ~ Incentive + Accuracy*Evidence + (1+Incentive|Subject)	33767	33824	4 vs. 5	51.92	<0.001	5
**6**	Confidence ~ Incentive + Accuracy*Evidence + Gender + (1+Incentive|Subject)	33769	33833	5 vs. 6	0.006	0.936	5
**7**	Confidence ~ Incentive + Accuracy*Evidence + Age + (1+Incentive|Subject)	33769	33833	5 vs. 7	0.16	0.687	5
**8**	Confidence ~ Incentive + Accuracy*Evidence + Difficulty + (1+Incentive|Subject)	33788	33808	5 vs. 8	33.11	<0.001	8
9	Confidence ~ Incentive + RT + Accuracy*Evidence + Difficulty + (1+Incentive|Subject)	33142	33218	8 vs. 9	598.25	<0.001	9

Supplementary Table 1: Model descriptions and comparison

Shown here are the model notations of all nine models with their respective AIC and BIC values, as well as model comparisons with corresponding χ^2 and P-values, resulting in the winning model 9.

Full Behavioral Results<br>*Confidence ~ Incentive + RT + Accuracy*Evidence + Difficulty + (1+Incentive|Subject)*	
Intercept (B0)	β = 76.56 ± 1.27<br>t_{45} = 60.36<br>P < 2e-16
Incentive	β = 0.88 ± 0.30<br>t_{32} = 2.94<br>P = 0.006
RT	β = -5.24 ± 0.21<br>t_{4305} = -25.34<br>P < 2e-16
Accuracy	β = 3.30 ± 0.42<br>t_{4290} = 7.86<br>P = 4.71e-15
Accuracy * Evidence	β = 2.83 ± 0.50<br>t_{4275} = 5.69<br>P = 1.38e-08
Difficulty hard	β = -2.22 ± 0.43<br>t_{4258} = -5.20<br>P = 2.07e-07
Difficulty medium	β = -1.53 ± 0.41<br>t_{4256} = -3.71<br>P = 0.0002

Supplementary Table 2: Results of linear mixed-effects model

Shown here are the results of the full linear mixed-effects model of the winning model. β: estimated regression coefficients for fixed effects ± estimated standard error of the regression coefficients, with corresponding t- and P-values.

Supplementary Note 2: Explorative analyses VS

We also applied our ROI analytical strategy to the VS. Like for the VMPFC and dACC analyses we built an independent anatomical ROI of the VS from the Brainnetome Atlas[4] (Supplementary Figure 1a).

We compared early certainty, incentive and confidence-related activations during both time-points in all available GLMs within the VS ROI (see Figure 4 in main text for comparable analysis in VMPFC). Thus, we extracted individual standardized regression coefficients (t-values) from the VS, corresponding to these respective activations and statistically compared them using repeated measure ANOVAs and post-hoc paired t-tests (Supplementary Figure 1, Supplementary Table 3). Activations for early certainty during choice moment were similar for all GLMs (ANOVA $F(4,29)= 0.43$, $P=0.787$; Supplementary Figure 1b), and was only positively related to early certainty in GLM2b (but marginally positively related in all other GLMs) (GLM1: $t_{29}= 2.01$, $P = 0.0541$; GLM2a: $t_{29}= 2.01$, $P = 0.0536$; GLM2b: $t_{29}= 2.12$, $P = 0.0428$; GLM3: $t_{29}= 2.00$, $P = 0.0531$; GLM4: $t_{29}= 2.00$, $P = 0.0547$). GLM specification had an impact on the incentive activation (ANOVA, main effect of GLM; $F(3,29) = 9.28$, $P < 0.001$; Supplementary Figure 1c), but not on the confidence activations (ANOVA, main effect of GLM; $F(3,29) = 1.37$, $P = 0.2561$; Supplementary Figure 1d) during incentive/rating moment. In the incentive case, post-hoc t-tests showed that T-values extracted from the GLM3 that related to the EV regressor were significantly higher than from other GLMs with a different coding of incentives (GLM1 versus GLM3: $t_{29}= -3.39$, $P = 0.002$; GLM2b versus GLM3: $t_{29}= -3.62$, $P = 0.001$; GLM4 versus GLM3: $t_{29} = -3.75$, $P<0.001$), but activity related to EV and confidence or certainty during rating moment were found to be similarly strong.

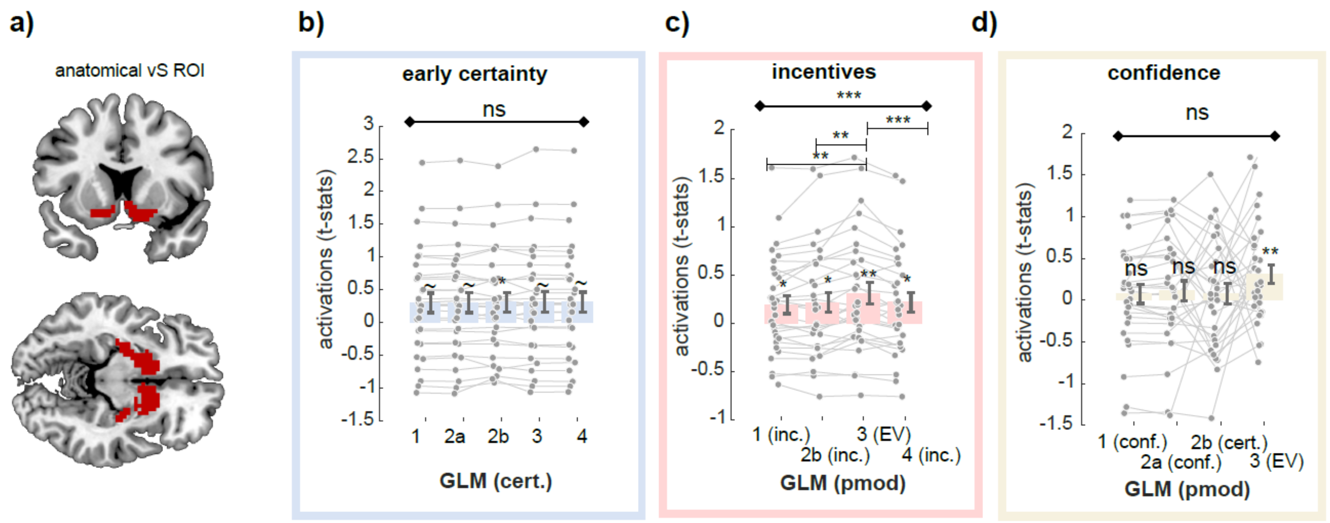

Supplementary Figure 1: Activation in Ventral Striatum Across Models
a) Anatomical VS region of interest (ROI). **b-d)** Comparison of dACC activations to different specifications of early certainty during choice moment (B), incentives during incentive/rating moment (C) and confidence during incentive/rating moment (D), as implemented in the different GLMs. Dots represent individual activations (N=30); bar and error bars indicate sample mean ± standard error of the mean. Grey lines highlight within subject variation across the different specifications. Cert: early certainty; Inc.: incentives; conf: confidence; EV: expected value; Diamond-ended horizontal bars indicate the results of repeated-measure ANOVAs. Dash-ended horizontal bars indicate the result of post-hoc paired t-tests. ~ P < 0.10; * P<0.05; ** P<0.01; *** P < 0.001. For repeated-measure ANOVA results: ns P>0.05, for one-sample t-tests: ns P>0.1.

Finally, we repeated the qualitative falsification exercise (see Figure 5 in the main text) for the VS ROI. We extracted the VS activations for all regressors in GLM5 using our ROI, and compared them with the theorized qualitative patterns (Supplementary Figure 2, Supplementary Table 4-5). At the stimulus/choice moment, we found no effect of incentive conditions on dACC baseline activity, nor on its correlation with confidence – "slope" (ANOVA baseline: P = 0.9616; ANOVA slope: P = 0.2595). At rating moment, incentive conditions had an effect on VS baseline activity (ANOVA F(2,29)= 6.40, P= 0.0031). Post-hoc testing revealed that VS baseline activity was significantly positive in all incentive conditions (Loss: t_{29} = 5. 26, P <0.001 ; Neutral: t_{29} = 3.37, P = 0.022; Gain: t_{29} = 6.17, P<0.001), but larger in gain versus loss (t_{29} = -2.20, P = 0.036) and in gain vs neutral conditions (t_{29} = -2.93, P = 0.006), but not in loss vs neutral condition (t_{29} = 1.87, P = 0.072) (see Supplementary Table 4-5). Incentive conditions had a significant effect (ANOVA F(2,29)= 5.94 P = 0.005) on the slope of the correlation of VS activity with confidence, where

only in the gain condition the slope was positive (t_{29} = 2.79, P = 0.009). Post-hoc testing showed that the correlation with confidence was significantly higher in gain versus loss (t_{29} = -3.16, P = 0.0036), and higher for gain versus neutral conditions (t_{29} = -2.72, P = 0.0109), whereas no difference was found for neutral versus loss condition (t_{29} = 0.41, P = 0.688). Again, similar to the results in the VMPFC and dACC, the observed pattern of VS activity was not featured in the EV model, nor in the confidence model, or any other model prediction, and thus points to a more complex picture of disruption of metacognitive signals due to motivational signals.

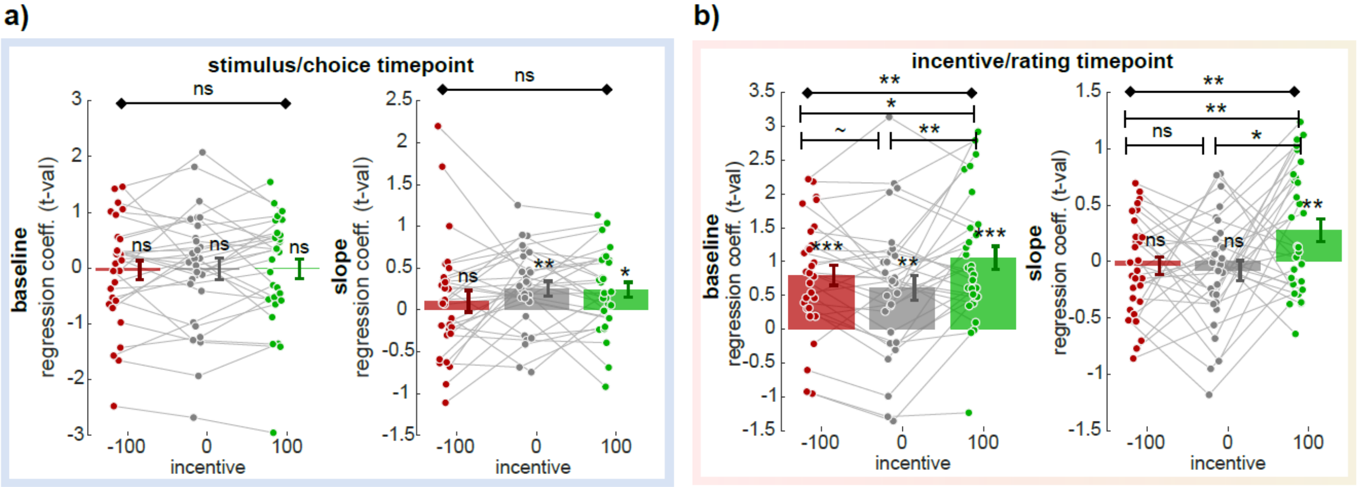

Supplementary Figure 2: Activation in Ventral Striatum across Incentives and Timepoints
a-b) VS ROI analysis. T-values corresponding to baseline and regression slope were extracted in the three incentive conditions, and at the two time-points of interest (A: stimulus/choice; B: incentive/rating). Dots represent individual activations (N=30); bar and error bars indicate sample mean ± standard error of the mean. Grey lines highlight within subject variation across the different incentive conditions. Diamond-ended horizontal bars indicate the results of repeated-measure ANOVAs. Dash-ended horizontal bars indicate the result of post-hoc paired t-tests. ~ $P < 0.10$; * $P<0.05$; ** $P<0.01$; *** $P < 0.001$. For repeated-measure ANOVA results: ns $P>0.05$, for one-sample t-tests: ns $P>0.1$.

**Early certainty**	**GLM1**	**GLM2a**	**GLM2b**	**GLM3**	**GLM4**
	0.30 ± 0.15<br>t_{29} = 2.0075<br>P = 0.0541	0.30 ± 0.15<br>t_{29} = 2.0120<br>P = 0.0536	0.31 ± 0.15<br>t_{29} = 2.1190<br>P = 0.0428	0.31 ± 0.16<br>t_{29} = 2.0162<br>P = 0.0531	0.31 ± 0.15<br>t_{29} = 2.0025<br>P = 0.0547
	**ANOVA (Main effect of GLM)**	**-**	**-**	**-**	**-**
	F(4,29)=0.43<br>P=0.7870	-	-	-	
**Incentive**		**GLM1**	**GLM2b**	**GLM3**	**GLM4**
		0.1915 ± 0.0926<br>t_{29} = 2.0684<br>P = 0.0476	0.2159 ± 0.1040<br>t_{29} = 2.0750<br>P = 0.0470	0.3095 ± 0.1113<br>t_{29} = 2.7793<br>P = 0.0095	0.2166 ± 0.1010<br>t_{29} = 2.1448<br>P = 0.0405
	**ANOVA (Main effect of GLM)**	**T-Test (3 vs 1)**	**T-Test (3 vs 2b)**	**-**	**T-Test (3 vs 4)**
	F(3,29)= 9.28<br>P=2.17206e-05	-0.1180 ± 0.0348<br>t_{29} = -3.3930<br>P = 0.0020	-0.0936 ± 0.0258<br>t_{29} = -3.6234<br>P = 0.0011	-	-0.0928 ± 0.0248<br>t_{29} = -3.7459<br>P = 7.9381e-04
**Confidence**		**GLM1**	**GLM2a**	**GLM2b**	**GLM3**
		0.0742 ± 0.1120<br>t_{29} = 0. 6626<br>P = 0. 5128	0.1096 ± 0.1221<br>t_{29} = 0. 8974<br>P = 0. 3769	0.0770 ± 0.1229<br>t_{29} = 0. 6265<br>P = 0. 5359	0.3095 ± 0.1113<br>t_{29} = 2. 7793<br>P = 0. 0095
	**ANOVA (Main effect of GLM)**	-	-	-	**-**
	F(3,29) = 1.37<br>P = 0.2561	-	-	-	-

Supplementary Table 3: Comparison of VS parametric activity (t-values) as a function of model specification (GLMs)

The table reports descriptive and inferential statistics on VS ROI parametric activations with three different variables of interest: early certainty effects at choice moment, incentive effects at rating moment and confidence effects at rating moment (see **Figure S5**). Per effect of interest, results of one-sample t-tests against zero, repeated-measure (RM) ANOVAs on the main effect of GLMS, and post-hoc t-test results are shown.

Choice/Stim	baseline	**Inc. -100**	**Inc. 0**	**Inc. +100**	**ANOVA**
		-0.04 ± 0.17<br>t_{29} = -0.2118<br>P = 0. 8337	-0.01 ± 0.19<br>t_{29} = -0.0692<br>P = 0. 9453	-0.01 ± 0.17<br>t_{29} = -0.0481<br>P = 0. 9620	F(2,29) = 0.04<br>P = 0.9616
	slope	**Inc. -100**	**Inc 0**	**Inc. +100**	**ANOVA**
		0.10 ± 0.13<br>t_{29} = 0.8188<br>P = 0.4196	0.26 ± 0.09<br>t_{29} = 2.9434<br>P = 0.0063	0.24 ± 0.09<br>t_{29} = 2.6902<br>P = 0.0117	F(2,29) = 1.38<br>P = 0.2595

Supplementary Table 4: Comparison of VS activity at choice moment (t-values), as a function of incentive condition

The table reports descriptive and inferential statistics on VS ROI parametric activations in our three incentive conditions during choice moment, for both baseline activity as well as the correlation with early certainty (i.e. slope) (see **Figure S6**). Results of RM ANOVAs and one-sample t-tests against 0 are shown.

**Incentive/rating**	**baseline**	**Inc -100**	**Inc 0**	**Inc +100**	**ANOVA**
		0.80 ± 0.15<br>t_{29} = 5.2603<br>P = 1.2305e-05	0.61 ± 0.18<br>t_{29} = 3.3655<br>P = 0.0022	1.06 ± 0.17<br>t_{29} = 6.1747<br>P = 9.8752e-07	F(2,29) = 6.40<br>P = 0.0031
		**T-Test**<br>[-100 vs 0]	**T-Test**<br>[0 vs 100]	**T-Test**<br>[-100 vs 100]	
		0.19 ± 0.10<br>t_{29} = 1.8707<br>P = 0.0715	-0.45 ± 0.15<br>t_{29} = -2.9268<br>P = 0.0066	-0.26 ± 0.12<br>t_{29} = -2.1995<br>P = 0.0360	
	**slope**	**Inc -100**	**Inc 0**	**Inc +100**	**ANOVA**
		-0.04 ± 0.08<br>t_{29} = -0.4695<br>P = 0.6422	-0.08 ± 0.09<br>t_{29} = -0.9138<br>P = 0.3684	0.28 ± 0.10<br>t_{29} = 2.7922<br>P = 0.0092	F(2,29) = 5.94<br>P = 0.0045
		**T-Test**<br>[-100 vs 0]	**T-Test**<br>[0 vs 100]	**T-Test**<br>[-100 vs 100]	
		0.04 ± 0.11<br>t_{29} = 0.41<br>P = 0.6877	-0.36 ± 0.13<br>t_{29} = -2.7197<br>P = 0.0109	-0.32 ± 0.10<br>t_{29} = -3.1642<br>P = 0.0036	

Supplementary Table 5: Comparison of VS activity at rating moment (t-values), as a function of incentive condition

The table reports descriptive and inferential statistics on VS ROI parametric activations in our three incentive conditions during rating moment, for both baseline activity as well as the correlation with confidence (i.e. slope) (see **Figure S6**). Results of one-sample t-tests against 0, RM ANOVAs and post-hoc t-tests are shown.

Supplementary Note 3: Explorative analyses dACC

Explorative analysis of dACC results show overlap between confidence and EV signal

While we did not find clear evidence for VMPFC activity correlating with confidence at our pre-specified statistical threshold, we did find a cluster of dACC activity positively correlating with both confidence (Figure 2a) and EV (Figure 2b). We therefore applied our ROI analytical strategy – originally designed for the VMPFC – to the dACC. Like for the VMPFC analyses we built an independent anatomical ROI of the dACC from the Brainnetome Atlas[4] (Supplementary Figure 2a).

We compared early certainty, incentive and confidence-related activations during both time-points in all available GLMs within the dACC ROI (see Figure 4 in main text for comparable analysis in VMPFC). Thus, we extracted individual standardized regression coefficients (t-values) from the dACC, corresponding to these respective activations and statistically compared them using repeated measure ANOVAs and post-hoc paired t-tests (Supplementary Figure 2, Supplementary Table 6). Activations for early certainty during choice moment were similar for all GLMs (ANOVA $F(4,29)= 1.75$, $P=0.149$; Supplementary Figure 2b), and all were significantly negatively related to early certainty (GLM1: $t_{29}= -2.48$, $P = 0.019$; GLM2a: $t_{29}= -2.48$, $P = 0.019$; GLM2b: $t_{29}= -2.39$, $P = 0.024$; GLM3: $t_{29}= -2.48$, $P = 0.019$; GLM4: $t_{29}= -2.51$, $P = 0.018$). GLM specification had an impact on the incentive activation (ANOVA, main effect of GLM; $F(3,29) = 19.13$, $P < 0.001$; Supplementary Figure 3c), but not on the confidence activations (ANOVA, main effect of GLM; $F(3,29) = 1.95$, $P = 0.127$; Supplementary Figure 3d) during incentive/rating moment. In the incentive case, post-hoc t-tests showed that T-values extracted from the GLM3 that related to the EV regressor were significantly higher than from other GLMs with a different coding of incentives (GLM1 versus GLM3: $t_{29}= -5.22$, $P < 0.001$; GLM2b versus GLM3: $t_{29}= -4.45$, $P<0.001$; GLM4 versus GLM3: $t_{29} = -4.31$, $P<0.001$), but activity related to EV and confidence or certainty during rating moment were found to be similarly strong.

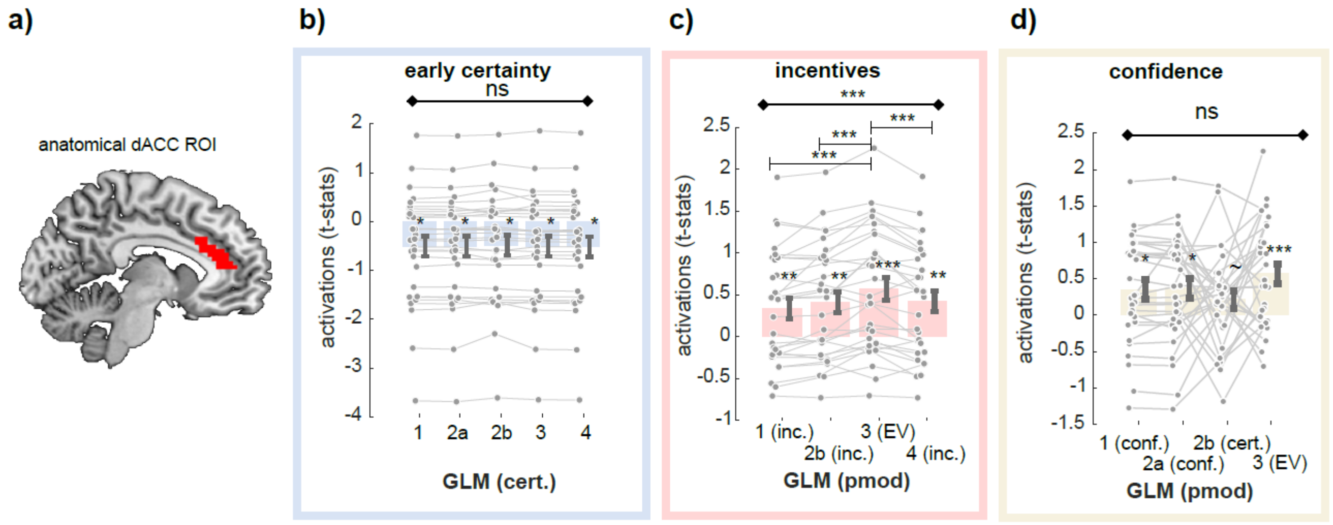

Supplementary Figure 3: Activation in Dorsal Anterior Cingulate Cortex Across Models
a) Anatomical dACC region of interest (ROI). **b-d)** Comparison of dACC activations to different specifications of early certainty during choice moment (B), incentives during incentive/rating moment (C) and confidence during incentive/rating moment (D), as implemented in the different GLMs. Dots represent individual activations (N=30); bar and error bars indicate sample mean ± standard error of the mean. Grey lines highlight within subject variation across the different specifications. Cert: early certainty; Inc.: incentives; conf: confidence; EV: expected value; Diamond-ended horizontal bars indicate the results of repeated-measure ANOVAs. Dash-ended horizontal bars indicate the result of post-hoc paired t-tests. ~ $P < 0.10$; * $P<0.05$; ** $P<0.01$; *** $P < 0.001$. For repeated-measure ANOVA results: ns $P>0.05$, for one-sample t-tests: ns $P>0.1$.

Finally, we repeated the qualitative falsification exercise (see Figure 5 in the main text) for the dACC ROI. We extracted the dACC activations for all regressors in GLM5 using our ROI, and compared them with the theorized qualitative patterns (Supplementary Figure 3, Supplementary Table S7-8). At the stimulus/choice moment, we found no effect of incentive conditions on dACC baseline activity, nor on its correlation with confidence – "slope" (ANOVA baseline: $P = 0.952$; ANOVA slope: $P = 0.534$). At rating moment, incentive conditions had an effect on dACC baseline activity (ANOVA $F(2,29)= 12.30$, $P<0.001$). Post-hoc testing revealed that dACC baseline activity was significantly positive in all incentive conditions (Loss: $t_{29} = 3.96$, $P <0.001$; Neutral: $t_{29} = 2.69$, $P = 0.011$; Gain: $t_{29} = 6.31$, $P<0.001$), but larger in gain versus loss ($t_{29} = -3.63$, $P = 0.001$) and in gain vs neutral conditions ($t_{29} = -4.10$, $P < 0.001$), but not in loss vs neutral condition ($t_{29} = 1.71$, $P = 0.098$) (see Supplementary Table S7-8). Incentive conditions had a marginally significant effect (ANOVA $F(2,29)= 3.12$ $P = 0.052$) on the slope of the correlation of dACC activity with confidence,

where only in the gain condition the slope was positive (t_{29} = 3.35, P = 0.002). Post-hoc testing showed that the correlation with confidence was only significantly higher in gain versus loss (t_{29} = -2.37, P = 0.025), and marginally higher for gain versus neutral conditions (t_{29} = -1.95, P = 0.060), whereas no difference was found for neutral versus loss condition (t_{29} = -0.18, P = 0.860). Again, similar to the results in the VMPFC, the observed pattern of dACC activity was not featured in the EV model, nor in the confidence model, or any other model prediction, and thus points to a more complex picture of disruption of metacognitive signals due to motivational signals.

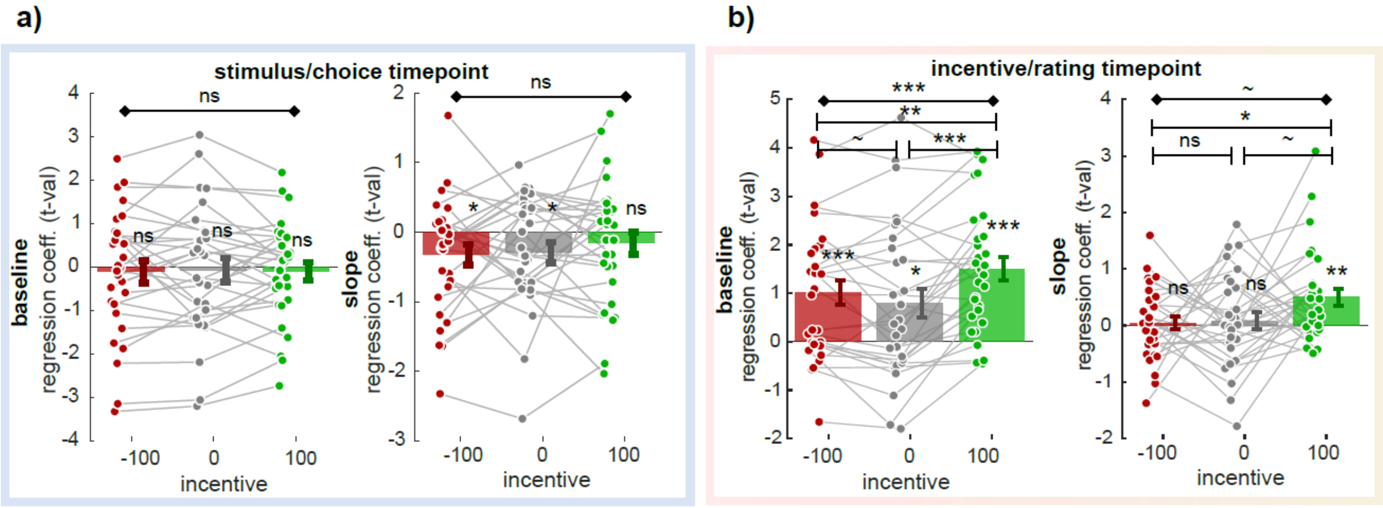

Supplementary Figure 4: Activation in Dorsal Anterior Cingulate Cortex across Incentives and Timepoints

a-b) dACC ROI analysis. T-values corresponding to baseline and regression slope were extracted in the three incentive conditions, and at the two time-points of interest (A: stimulus/choice; B: incentive/rating). Dots represent individual activations (N=30); bar and error bars indicate sample mean ± standard error of the mean. Grey lines highlight within subject variation across the different incentive conditions. Diamond-ended horizontal bars indicate the results of repeated-measure ANOVAs. Dash-ended horizontal bars indicate the result of post-hoc paired t-tests. ~ P < 0.10; * P<0.05; ** P<0.01; *** P < 0.001. For repeated-measure ANOVA results: ns P>0.05, for one-sample or two sample t-tests: ns P>0.1.

**Early certainty**	**GLM1**	**GLM2a**	**GLM2b**	**GLM3**	**GLM4**
	-0.5 ± 0.2<br>t_{29} = -2.48<br>P = 0.019	-0.51 ± 0.2<br>t_{29} = -2.48<br>P = 0.019	-0.48 ± 0.2<br>t_{29} = -2.39<br>P = 0.024	-0.51 ± 0.21<br>t_{29} = -2.48<br>P = 0.019	-0.52 ± 0.21<br>t_{29} = -2.51<br>P = 0.018
	**ANOVA (Main effect of GLM)**	**-**	**-**	**-**	**-**
	F(4,29)=1.75<br>P=0.1439	-	-	-	
**Incentive**		**GLM1**	**GLM2b**	**GLM3**	**GLM4**
		0.34 ± 0.12<br>t_{29} = 2. 82<br>P = 0. 0085	0.41 ± 0.12<br>t_{29} = 3. 34<br>P = 0. 0023	0.57 ± 0.13<br>t_{29} = 4. 25<br>P = 0. 0002	0.42 ± 0.12<br>t_{29} = 3. 43<br>P = 0. 0018
	**ANOVA (Main effect of GLM)**	**T-Test (3 vs 1)**	**T-Test (3 vs 2b)**	**-**	**T-Test (3 vs 4)**
	F(3,29)=19.13<br>P= 1.0e-08	-0.23 ± 0.09<br>t_{29} = -5.22<br>P = 1.3781e-05	-0.16 ± 0.07<br>t_{29} = -4.45<br>P = 1.1638e-04	-	-0.15 ± 0.07<br>t_{29} = -4.3<br>P = 1.7082e-04
**Confidence**		**GLM1**	**GLM2a**	**GLM2b**	**GLM3**
		0. 35 ± 0.13<br>t_{29} = 2. 65<br>P = 0. 0128	0.37 ± 0.14<br>t_{29} = 2. 75<br>P = 0. 0102	0.22 ± 0.12<br>t_{29} = 1.82<br>P = 0. 0795	0.57 ± 0.13<br>t_{29} = 4. 25<br>P = 0. 0002
	**ANOVA (Main effect of GLM)**	**T-Test (3 vs 1)**	**T-Test (3 vs 2a)**	**T-Test (3 vs 2b)**	**-**
	F(3,29) = 1.95<br>P = 0.1272	-0.22 ± 0.36<br>t_{29} = -1. 24<br>P = 0. 2257	-0.20 ± 0.35<br>t_{29} = -1.15<br>P =0.2583	-0.35 ± 0.33<br>t_{29} = -2.20<br>P = 0. 036	

Supplementary Table 6: Comparison of ACC parametric activity (t-values) as a function of model specification (GLMs)

The table reports descriptive and inferential statistics on ACC ROI parametric activations with three different variables of interest: early certainty effects at choice moment, incentive effects at rating moment and confidence effects at rating moment (see **Figure S3**). Per effect of interest, results of one-sample t-tests against zero, repeated-measure (RM) ANOVAs on the main effect of GLMS, and post-hoc t-test results are shown.

Choice/Stim		Inc. -100	Inc. 0	Inc. +100	ANOVA
	**baseline**	-0.11 ± 0.26<br>t_{29} = -0.43<br>P = 0.67	-0.07 ± 0.27<br>t_{29} = -0.26<br>P = 0.80	-0.10 ± 0.21<br>t_{29} = -0.49<br>P = 0.63	F(2,28)<br>= 0.05<br>P = 0.95
		**Inc. -100**	**Inc 0**	**Inc. +100**	**ANOVA**
	**slope**	-0.33 ± 0.15<br>t_{29} = -2.17<br>P = 0.04	-0.29 ± 0.15<br>t_{29} = -1.98<br>P = 0.06	-0.16 ± 0.16<br>t_{29} = -0.98<br>P = 0.34	F(2,28)<br>= 0.63<br>P = 0.53

Supplementary Table 7: Comparison of ACC activity at choice moment (t-values), as a function of incentive condition

The table reports descriptive and inferential statistics on ACC ROI parametric activations in our three incentive conditions during choice moment, for both baseline activity as well as the correlation with early certainty (i.e. slope) (see **Figure S4**). Results of RM ANOVAs and one-sample t-tests against 0 are shown.

<table>
<tr><td rowspan="8">Incentive/rating</td><td rowspan="4">baseline</td><td>Inc -100</td><td>Inc 0</td><td>Inc +100</td><td>ANOVA</td></tr>
<tr><td>1.01 ± 0.25
$t_{29} = 3.96$
P = 0.0004</td><td>0.79 ± 0.29
$t_{29} = 2.69$
P = 0.0117</td><td>1.50 ± 0.24
$t_{29} = 6.31$
$P = 6.83\times10^{-7}$</td><td>$F(2,28) = 12.30$
$P = 3.52\times10^{-5}$</td></tr>
<tr><td>T-Test
[-100 vs 0]</td><td>T-Test
[0 vs 100]</td><td>T-Test
[-100 vs 100]</td><td rowspan="2"></td></tr>
<tr><td>0.22 ± 0.13
$t_{29} = 1.71$
P = 0.0984</td><td>-0.71 ± 0.17
$t_{29} = -4.10$
$P = 3.01\times10^{-4}$</td><td>-0.49 ± 0.14
$t_{29} = -3.63$
P = 0.0011</td></tr>
<tr><td rowspan="4">slope</td><td>Inc -100</td><td>Inc 0</td><td>Inc +100</td><td>ANOVA</td></tr>
<tr><td>0.05 ± 0.12
$t_{29} = 0.41$
P = 0.68</td><td>0.08 ± 0.15
$t_{29} = 0.22$
P = 0.58</td><td>0.50 ± 0.15
$t_{29} = 3.35$
P = 0.0022</td><td>$F(2,28) = 3.12$
P = 0.0517</td></tr>
<tr><td>T-Test
[-100 vs 0]</td><td>T-Test
[0 vs 100]</td><td>T-Test
[-100 vs 100]</td><td rowspan="2"></td></tr>
<tr><td>-0.04 ± 0.20
$t_{29} = -0.18$
P = 0.86</td><td>-0.42 ± 0.21
$t_{29} = -1.95$
P = 0.06</td><td>-0.45 ± 0.19
$t_{29} = -2.37$
P = 0.0246</td></tr>
</table>

Supplementary Table 8: Comparison of ACC activity at rating moment (t-values), as a function of incentive condition

The table reports descriptive and inferential statistics on ACC ROI parametric activations in our three incentive conditions during rating moment, for both baseline activity as well as the correlation with confidence (i.e. slope) (see **Figure S4**). Results of one-sample t-tests against 0, RM ANOVAs and post-hoc t-tests are shown.

Supplementary Note 4: Additional Behavioral Analyses: Properties of Confidence Judgments

Similarly to Lebreton et al. (2018)[5], we performed additional behavioral analyses to confirm three main properties of confidence judgements, as theorized in a recent paper by Sanders and colleagues[6]. There, the authors outlined three main properties of confidence judgments, which should be observed if participants compute the probability of a choice being correct given some level of noisy evidence: (1) confidence ratings correlate with the probability of being correct; (2) the link between confidence ratings and evidence is positive for correct and negative for incorrect responses; (3) the link between evidence and performance differs between high and low confidence trials.

To assess the first property, we sorted trials according to the confidence ratings at the individual level. Then, we averaged trials over 8 bins per participant, and computed the frequency of correct choices in each bin. Finally, the correlation between the bins' confidence and performance was computed at the individual level. These measures were positively correlated ($R = 0.59 \pm 0.05$; Supplementary Figure 4a).

To assess the second property, the following linear regression was estimated at the individual level, using all trials from the confidence elicitation task (Model 1):

(1) $\text{Conf} = \beta 0 + \beta 1 \times \text{Correct} \times \text{Evidence} + \beta 2 \times \text{Incorrect} \times \text{Evidence}$,

where Incorrect is a dummy variable coding for incorrect answers, and Correct is a dummy variable coding for correct answers. Then, we tested the parameters of this model at the population level using one-sample t-tests. The results (Supplementary Figure 4b), summarized in the table below (Supplementary Table 9), demonstrate that confidence judgments are indeed positively associated with evidence for correct trials, and negatively for incorrect trials.

To assess the third property, we proceeded similarly to the second: the following logistic regression was estimated at the individual level, using all trials (Model 2):

(2) $\text{Correct} = \beta 0 + \beta 1 \times \text{High} \times \text{Evidence} + \beta 2 \times \text{Low} \times \text{Evidence}$,

where High is a dummy variable coding for high confidence trials (i.e. confidence > median(confidence)), and Low is a dummy variable coding for low confidence trials (i.e. confidence ≤ median(confidence)). Then, the parameters of this model were tested at the population level, using one-sample t-tests. The results

(Supplementary Figure 4c), summarized in the table below (Supplementary Table 9), indeed demonstrate that the curve has a steeper slope in the high than in the low confidence trials, as was expected.

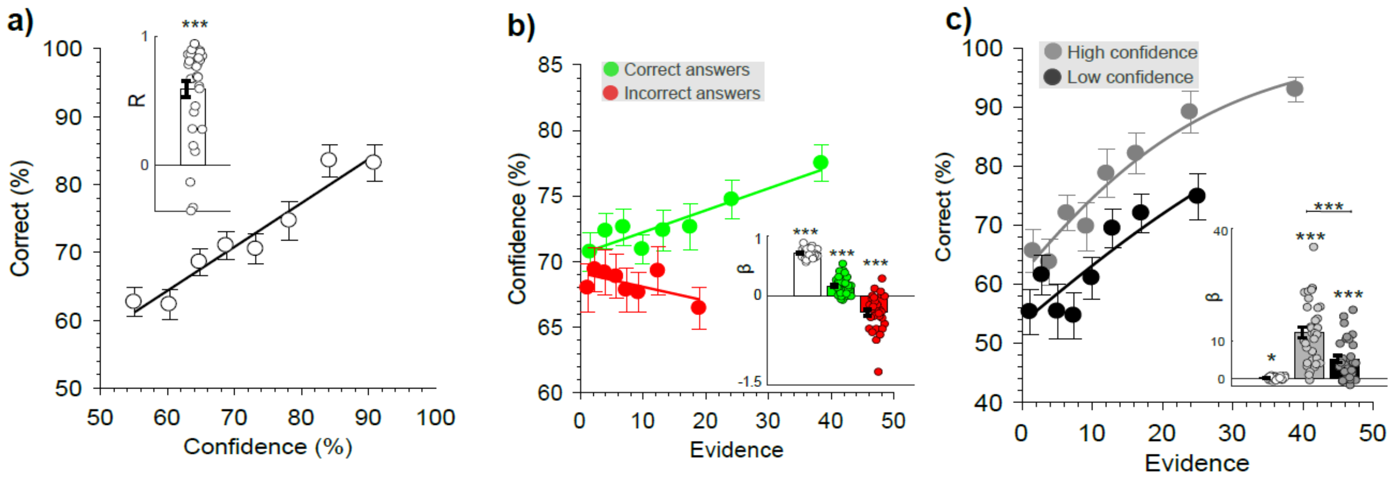

Supplementary Figure 5: Properties of Confidence Judgments

a) observed performance (% correct choices) as a function of reported confidence. **b)** reported confidence as function of evidence for correct (green) and incorrect (red) choices. **c)** observed performance (% correct choices) as a function of evidence, for high (gray) and low (black) confidence trials. The insets presented on the side of each graph depict the results of the population-level analyses on the correlation coefficients (a) or on the regression coefficients (b and c). Error bars indicate inter-subject standard errors of the mean. N = 32. *: P<.05; **: P<.01; ***P<.001

**Model 1 (Figure S1b)**	
**Intercept (β_0)**	$\beta = 0.71 \pm 0.01$<br>$t_{31} = 53.06$<br>P = 5.3528e-32
**Confidence/Evidence Correct Answers (β_1)**	$\beta = 0.16 \pm 0.03$<br>$t_{31} = 5.86$<br>P = 1.8326e-06
**Confidence/Evidence Incorrect Answers (β_2)**	$\beta = -0.28 \pm 0.05$<br>$t_{31} = -5.36$<br>P = 7.7032e-06
**Model 2 (Figure S1c)**	
**Intercept (β_0)**	$\beta = 0.14 \pm 0.07$<br>$t_{31} = 2.04$<br>P = 0.0495
**Performance/Evidence High confidence (β_1)**	$\beta = 12.23 \pm 1.49$<br>$t_{31} = 8.21$<br>P = 2.8097e-09
**Performance/Evidence Low confidence (β_2)**	$\beta = 5.14 \pm 0.93$<br>$t_{31} = 5.50$<br>P = 5.1270e-06
**Difference ($\beta_1 - \beta_2$)**	$t_{31} = 5.45$<br>P = 5.8544e-06

Supplementary Table 9: Results of linear mixed-effects models for properties of confidence judgments

Supplementary Note 5: Early certainty

In this section, we provide further details about the computation and properties of the early certainty variable. To verify that our model of early certainty is an appropriate proxy of confidence judgments, we performed similar behavioral analyses to confirm the three main properties of confidence judgments still hold for our early certainty variable. We performed identical analyses, substituting subjective confidence judgments for early certainty values.

Our results show that the measures of early certainty and performance are highly correlated ($R = 0.67 \pm 0.07$; Supplementary Figure 5a, Supplementary Table 10). Early certainty is also positively associated with evidence for correct trials, and negatively for incorrect trials (Supplementary Figure 5b, Supplementary Table 10). Finally, the relationship between performance and evidence is indeed higher in trials with high early certainty versus low early certainty (Supplementary Figure 5c, Supplementary Table 10).

When inspecting the beta values for the second model (Supplementary Figure 5c, Supplementary Table 10), we observed three statistical outliers (i.e. >1.5 times the interquartile range away from the 75th percentile) in the effect of evidence on performance in trials with high early certainty (β_1). These outliers were caused by the median-split of the early certainty trials into high and low variants, as these subjects performed (almost) perfectly in the high early certainty trials, causing the betas to inflate. Importantly, when excluding these subjects from the analyses, we found identical results, albeit stronger ($\beta_0 = 0.09 \pm 0.07$, $t_{28} = 1.26$, $P = 0.217$; $\beta_1 = 17.99 \pm 2.40$, $t_{28} = 7.49$, $P < 0.001$; $\beta_2 = 3.83 \pm 0.94$, $t_{28} = 4.06$, $P < 0.001$; Difference ($\beta_1 - \beta_2$): $t_{28} = 5.87$, $P<0.001$).

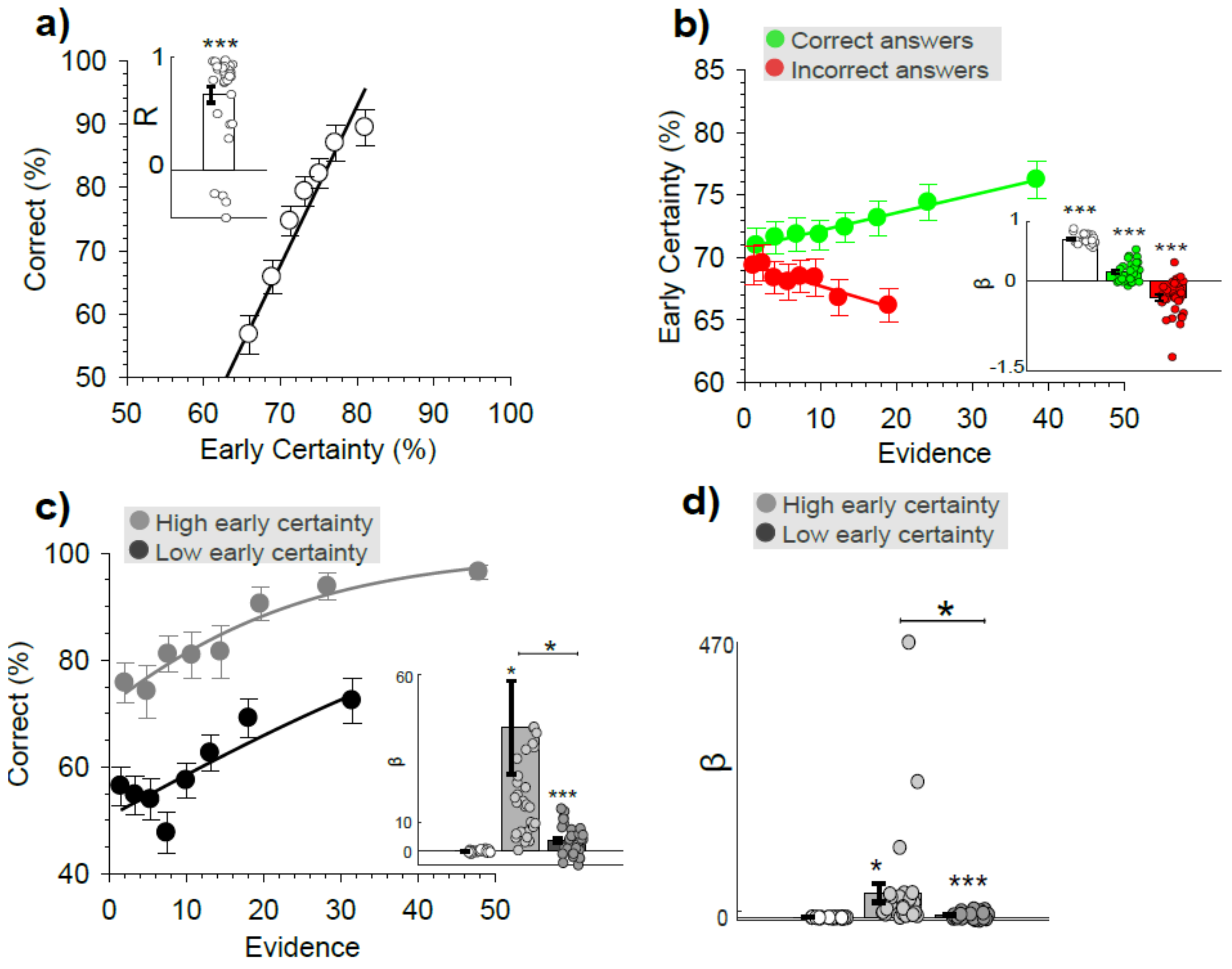

Supplementary Figure 6: Properties of Early Certainty

a) observed performance (% correct choices) as a function of early certainty. **b)** early certainty as function of evidence for correct (green) and incorrect (red) choices. **c)** observed performance (% correct choices) as a function of evidence, for high (gray) and low (black) early certainty trials. The insets presented on the side of each graph depict the results of the population-level analyses on the correlation coefficients (a) or on the regression coefficients (b and c), where dots represent individual correlation coefficients (a), or regression coefficients (b and c) (N=32); bar and error bars indicate sample mean ± inter-subject standard error of the mean. Main plots and insets in plot a and b include the three statistical outliers. **d)**: Shown here are the three statistical outliers for the individual regression coefficients for the high early certainty trials. For visibility we excluded those three outliers in the inset in plot c.

*: P<.05; **: P<.01; ***P<.001

Model 1 (Figure S2b)

**Intercept (β_0)**	$\beta = 0.70 \pm .01$<br>$t_{31} = 53.91$<br>P = 3.3040e-32
**Confidence/Evidence Correct Answers (β_1)**	$\beta = 0.15 \pm .03$<br>$t_{31} = 5.45$<br>P = 5.9907e-06
**Confidence/Evidence Incorrect Answers (β_2)**	$\beta = -0.27 \pm .05$<br>$t_{31} = -5.16$<br>P = 1.3702e-05
**Model 2 (Figure S2c)**	
**Intercept (β_0)**	$\beta = 0.11 \pm 0.07$<br>$t_{31} = 1.51$<br>P = 0.14
**Performance/Evidence High confidence (β_1)**	$\beta = 41.95 \pm 15.75$<br>$t_{31} = 2.67$<br>P = 0.0122
**Performance/Evidence Low confidence (β_2)**	$\beta = 3.64 \pm 0.90$<br>$t_{31} = 4.06$<br>P = 0.0003
**Difference ($\beta_1 - \beta_2$)**	$t_{31} = 2.40$<br>P = 0.0226

Supplementary Table 10: Results of linear mixed-effects models for properties of early certainty

Moreover, to validate that our model of early certainty correlates highly with subjective confidence and choice and stimulus features, but does not show a statistical relationship with incentives, we built a linear mixed-effects model using the lme4 package in R. We used early certainty as dependent variable and added RT, accuracy, evidence and the interaction between evidence and accuracy as predictors. Indeed, the results showed that RT, accuracy and the accuracy * evidence interaction all significantly contributed to early certainty, while no effect of incentive value on early certainty was found (Supplementary Table 11).

Early Certainty GLMER Results<br><br>*Early Certainty ~ Incentive + RT + Accuracy*Evidence + (1|Subject)*	
Intercept (B0)	$\beta = 75.52 \pm 1.15$<br>$t_{33} = 65.63$<br>P=<2e-16
Incentive	$\beta = 0.07 \pm 0.08$<br>$t_{4288} = 0.84$<br>P = 0.404
RT	$\beta = -5.69 \pm 0.08$<br>$t_{4292} = -71.90$<br>P < 2e-16
Accuracy	$\beta = 3.61 \pm 0.16$<br>$t_{4288} = 22.75$<br>P = <2e-16
Accuracy * Evidence	$\beta = 2.50 \pm 0.19$<br>$t_{4288} = 13.16$<br>P = <2e-16

Supplementary Table 11: Results of general linear mixed-effects model
Shown here are the results of the full linear mixed-effects model. β: estimated regression coefficients for fixed effects ± estimated standard error of the regression coefficients, with corresponding t- and P-values.

Supplementary References

1. Bates, D., Mächler, M., Bolker, B. M. & Walker, S. C. Fitting linear mixed-effects models using lme4. *J. Stat. Softw.* **67**, 1–48 (2015).
2. Schaalje, G. B., McBride, J. B. & Fellingham, G. W. Adequacy of approximations to distributions of test statistics in complex mixed linear models. *J. Agric. Biol. Environ. Stat.* **7**, 512–524 (2002).
3. Kuznetsova, A., Brockhoff, P. B. & Christensen, R. H. B. **lmerTest** Package: Tests in Linear Mixed Effects Models. *J. Stat. Softw.* **82**, 1–26 (2017).
4. Fan, L. *et al.* The Human Brainnetome Atlas: A New Brain Atlas Based on Connectional Architecture. *Cereb. Cortex* **26**, 3508–3526 (2016).
5. Lebreton, M. *et al.* Two sides of the same coin: Monetary incentives concurrently improve and bias confidence judgments. *Sci. Adv.* **4**, eaaq0668 (2018).
6. Sanders, J. I., Hangya, B. B. & Kepecs, A. Signatures of a Statistical Computation in the Human Sense of Confidence. *Neuron* **90**, 499–506 (2016).
