# Supplementary material for: Motivational signals disrupt metacognitive signals in the human ventromedial prefrontal cortex
Source: Commun Biol. 2022 Mar 18;5:244. doi: 10.1038/s42003-022-03197-z (PMC8933484; doi:10.1038/s42003-022-03197-z)
Supplement: Supplementary file 3 — Description of Additional Supplementary Files [file 42003_2022_3197_MOESM3_ESM.pdf]

Description of Additional Supplementary Files

File name: Supplementary Data 1
Description: GLM1 activation table Brain activations (whole brain analyses) of GLM1 and GLM3 showing activity related to early certainty at choice moment, as well as activity related to incentive, confidence and EV at incentive/rating moment. All wholebrain activation maps were thresholded using family-wise error correction for multiple correction (FWE) at cluster level (P FWE_clu < 0.05), with a voxel cluster-defining threshold of P

File name: Supplementary Data 2
Description: GLM1 activation table with exclusive motor mask Brain activations (whole brain analyses) of GLM1 showing activity related to early certainty at choice moment, as well as activity related to incentive and confidence at incentive/rating moment, exclusively masked for motor-related activity patterns using a Neurosynth mask. All whole-brain activation maps were thresholded using family-wise error correction for multiple correction (FWE) at cluster level (P FWE_clu < 0.05), with a voxel cluster-defining threshold of P<0.001 uncorrected. Activity that positively correlates to given variable is denoted by '+', whereas negative correlations are denoted by '-'.
