# Supplementary material for: Motivational signals disrupt metacognitive signals in the human ventromedial prefrontal cortex
Source: Commun Biol. 2022 Mar 18;5:244. doi: 10.1038/s42003-022-03197-z (PMC8933484; doi:10.1038/s42003-022-03197-z)
Supplement: Supplementary file 6 — Reporting Summary [file 42003_2022_3197_MOESM6_ESM.pdf]

nature portfolio

Corresponding author(s): M. Hoven

Last updated by author(s): Feb 1, 2022

Reporting Summary

Nature Portfolio wishes to improve the reproducibility of the work that we publish. This form provides structure for consistency and transparency in reporting. For further information on Nature Portfolio policies, see our Editorial Policies and the Editorial Policy Checklist.

Statistics

For all statistical analyses, confirm that the following items are present in the figure legend, table legend, main text, or Methods section.

n/a	Confirmed	
☐	☒	The exact sample size (*n*) for each experimental group/condition, given as a discrete number and unit of measurement
☐	☒	A statement on whether measurements were taken from distinct samples or whether the same sample was measured repeatedly
☐	☒	The statistical test(s) used AND whether they are one- or two-sided<br>*Only common tests should be described solely by name; describe more complex techniques in the Methods section.*
☐	☒	A description of all covariates tested
☐	☒	A description of any assumptions or corrections, such as tests of normality and adjustment for multiple comparisons
☐	☒	A full description of the statistical parameters including central tendency (e.g. means) or other basic estimates (e.g. regression coefficient) AND variation (e.g. standard deviation) or associated estimates of uncertainty (e.g. confidence intervals)
☐	☒	For null hypothesis testing, the test statistic (e.g. *F*, *t*, *r*) with confidence intervals, effect sizes, degrees of freedom and *P* value noted<br>*Give P values as exact values whenever suitable.*
☒	☐	For Bayesian analysis, information on the choice of priors and Markov chain Monte Carlo settings
☒	☐	For hierarchical and complex designs, identification of the appropriate level for tests and full reporting of outcomes
☒	☐	Estimates of effect sizes (e.g. Cohen's *d*, Pearson's *r*), indicating how they were calculated

Our web collection on statistics for biologists contains articles on many of the points above.

Software and code

Policy information about availability of computer code

Data collection	MATLAB 2018a
Data analysis	MATLAB 2020b, SPM12, RStudio 1.4.1106

For manuscripts utilizing custom algorithms or software that are central to the research but not yet described in published literature, software must be made available to editors and reviewers. We strongly encourage code deposition in a community repository (e.g. GitHub). See the Nature Portfolio guidelines for submitting code & software for further information.

Data

Policy information about availability of data

All manuscripts must include a data availability statement. This statement should provide the following information, where applicable:

- Accession codes, unique identifiers, or web links for publicly available datasets
- A description of any restrictions on data availability
- For clinical datasets or third party data, please ensure that the statement adheres to our policy

All source data needed to evaluate or reproduce the figures and analyses described in the paper and supplementary materials are available online at 'https://doi.org/10.6084/m9.figshare.19114406.v1'. Second level neuroimaging maps can be found at 'https://neurovault.org/collections/12221/'.

Field-specific reporting

Please select the one below that is the best fit for your research. If you are not sure, read the appropriate sections before making your selection.

☒ Life sciences ☐ Behavioural & social sciences ☐ Ecological, evolutionary & environmental sciences

For a reference copy of the document with all sections, see nature.com/documents/nr-reporting-summary-flat.pdf

Life sciences study design

All studies must disclose on these points even when the disclosure is negative.

Sample size	No sample-size calculation was done, because at the time no data was present that could give us a good estimate of the effect size. In pilot work, it was found that there were significant behavioral effects with 24 subjects, which is why we set our sample size to +- 30.
Data exclusions	Behavioral and imaging data were excluded on session level when accuracy was below 60%, or when subjects did not show sufficient variation in their confidence reports with a standard deviation <5%. Imaging data were excluded on a session level when head movements > 3.5mm .
Replication	We intended to replicate behavioral findings of an incentive effect on confidence judgments, and therefore we have used the exact same cognitive computer task. We have replicated all earlier findings.
Randomization	In this study we did not have experimental groups to randomize participants in to.
Blinding	Blinding was not relevant to this study, since no interventions took place.

Reporting for specific materials, systems and methods

We require information from authors about some types of materials, experimental systems and methods used in many studies. Here, indicate whether each material, system or method listed is relevant to your study. If you are not sure if a list item applies to your research, read the appropriate section before selecting a response.

Materials & experimental systems

n/a	Involved in the study	
☒	☐	Antibodies
☒	☐	Eukaryotic cell lines
☒	☐	Palaeontology and archaeology
☒	☐	Animals and other organisms
☐	☒	Human research participants
☒	☐	Clinical data
☒	☐	Dual use research of concern

Methods

n/a	Involved in the study	
☒	☐	ChIP-seq
☒	☐	Flow cytometry
☐	☒	MRI-based neuroimaging

Human research participants

Policy information about studies involving human research participants

Population characteristics	We included 18 females and 14 males between 18 and 58 year old (sd: 9.76)
Recruitment	Participants were recruited via previous studies, online databases and advertising in the community.
Ethics oversight	METC AMC

Note that full information on the approval of the study protocol must also be provided in the manuscript.

Magnetic resonance imaging

Experimental design

Design type	Event-related design
Design specifications	Each subject performed two sessions of the task, each consisting of 72 trials with 24 trials per incentive condition in a randomized order. Inter trial interval was a random time interval between 4500 and 6000 ms.
Behavioral performance measures	We measured reaction time to stimulus choice, confidence judgment, incentive condition and performance. To

Behavioral performance measures	establish that subjects were performing the task as expected we calculated average and standard deviations of performance within subjects, as well as their confidence judgments.

Acquisition

Imaging type(s)	functional
Field strength	3T
Sequence & imaging parameters	37 T2* EPI functional slices were acquired with a multi-echo sequence and interleaved scan sequence. With the following parameters: TR = 2.375, TEs = 9.0, 24.0, 43.8 ms, 3mm voxel size, 37 transverse slices, 3 mm slice thickness, 0.3 mm slice gap, each session contained 570 volumes.
Area of acquisition	whole brain

Diffusion MRI ☐ Used ☒ Not used

Preprocessing

Preprocessing software	SPM 12. Multi-echo scans were combined and realigned. Images were co-registered with T1 structural image and segmented for normalization to MNI space and smoothed using a 6mm Gausiann kernel.
Normalization	Data were normalized to MNI space
Normalization template	MNI
Noise and artifact removal	6 motion parameters were included in GLM for each subject
Volume censoring	ArtRepair software was used to reduce artifacts and repair outlier volumes using interpolation. No censoring was applied.

Statistical modeling & inference

Model type and settings	Unvariate analysis. First level models included moment of choice, moment of incentive and moment of feedback as stick functions, to which various parametric modulators were added in different GLMs. All contrasts were computed at subject level and taken to a group level mixed effect analysis on second level.
Effect(s) tested	Using one-sample t-test we tested, in various GLMs, both the positive and negative effects of the parametric modulators (1) certainty, (2) incentive, (3) confidence and (4) expected value on the whole brain.

Specify type of analysis: ☒ Whole brain ☐ ROI-based ☐ Both

Statistic type for inference (See Eklund et al. 2016)	All whole-brain activation maps were thresholded using family wise error correction for multiple comparisons (FWE) at cluster level, with a voxel cluster defining threshold of p<.001 uncorrected
Correction	FWE, see above

Models & analysis

n/a	Involved in the study
☒	☐ Functional and/or effective connectivity
☒	☐ Graph analysis
☒	☐ Multivariate modeling or predictive analysis
